# A smart thermoresponsive macroporous 4D structure by 4D printing of Pickering-high internal phase emulsions stabilized by plasma-functionalized starch nanomaterials for a possible delivery system

**DOI:** 10.1016/j.crfs.2024.100686

**Published:** 2024-02-01

**Authors:** Mahdiyar Shahbazi, Henry Jäger, Rammile Ettelaie, Jianshe Chen, Adeleh Mohammadi, Peyman Asghartabar Kashi, Marco Ulbrich

**Affiliations:** a Institute of Food Technology, University of Natural Resources and Life Sciences (BOKU), Muthgasse 18, 1190, Vienna, Austria; b Food Colloids and Bioprocessing Group, School of Food Science and Nutrition, University of Leeds, Leeds, LS2 9JT, UK; c Food Oral Processing Laboratory, School of Food Science & Biotechnology, Zhejiang Gongshang University, Hangzhou 310018, China; d Faculty of Food Science and Technology, Gorgan University of Agricultural Sciences and Natural Resources, Gorgan, 4913815739, Iran; e Faculty of Biosystem, College of Agricultural and Natural Resources, Tehran University, 31587-77871, Karaj, Iran; f Department of Food Technology and Food Chem., Chair of Food Process Engineering, Technische Universität Berlin, OfficeTK1, Ackerstraße 76, 13355, Berlin, Germany

**Keywords:** Pickering-HIPEs, 4D printing, Macroporous structures, LCST behavior, Hydrodynamic radius, Thermoresponsive behavior, Flocculated emulsion

## Abstract

Hierarchically porous structures combine microporosity, mesoporosity, and microporosity to enhance pore accessibility and transport, which are crucial to develop high performance materials for biofabrication, food, and pharmaceutical applications. This work aimed to develop a 4D-printed smart hierarchical macroporous structure through 3D printing of Pickering-type high internal phase emulsions (Pickering-HIPEs). The key was the utilization of surface-active (hydroxybutylated) starch nanomaterials, including starch nanocrystals (SNCs) (from waxy maize starch through acid hydrolysis) or starch nanoparticles (SNPs) (obtained through an ultrasound treatment). An innovative procedure to fabricate the functionalized starch nanomaterials was accomplished by grafting 1,2-butene oxide using a cold plasma technique to enhance their surface hydrophobicity, improving their aggregation, and thus attaining a colloidally stabilized Pickering-HIPEs with a low concentration of each surface-active starch nanomaterial. A flocculation of droplets in Pickering-HIPEs was developed after the addition of modified SNCs or SNPs, leading to the formation of a gel-like structure. The 3D printing of these Pickering-HIPEs developed a highly interconnected large pore structure, possessing a self-assembly property with thermoresponsive behavior. As a potential drug delivery system, this thermoresponsive macroporous 3D structure offered a lower critical solution temperature (LCST)-type phase transition at body temperature, which can be used in the field of smart releasing of bioactive compounds.

## Highlights

• Hydroxybutyl modification of SNP and SNC leads to hydrophobic thermoresponsive character.
• Pickering-HIPEs formulated by modified SNP and SNC offer a thixotropic property with recoverable matrix.
• The droplets in Pickering-HIPEs existed in flocculated shapes as shown by CLSM.
• 3D-printed samples revealed a hierarchically porous structure with a thermoresponsive behavior.

## Graphical abstract

**Figure:**
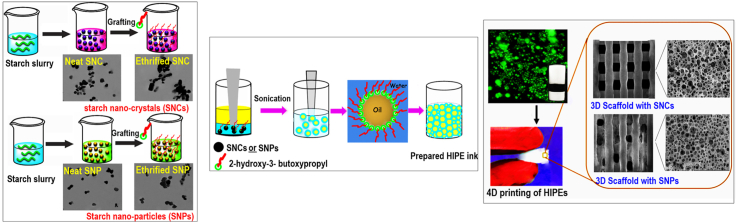

## 1 Introduction

The developments of advanced smart materials with high levels of porosity and high surface area have gained considerable attention because of their extensive range of applications in water treatment (Yang and Liu, 2019; Huang et al., 2022), tissue engineering (Osathanon et al., 2008; Paljevac et al., 2018), medical diagnostics and biosensors (Abdelmoaty et al., 2018; Zhang, Li, & Wang, 2020), controlled drug delivery (Grant et al., 2010; Bhunia et al., 2014), biomedical (Grant et al., 2010; Dong et al., 2014), and food science (Zhou et al., 2019; Wu et al., 2021). Principally, a spherical cavity in a porous structure is denoted as ‘voids’ (Cameron, 2005) or ‘cell’ (Silverstein, 2014) to describe HIPEs' pore features, which belong to the class of open-cell (solid). Next, an interconnecting pore among each void/cell and its neighbor is known as a ‘windows’. Lastly, a much smaller pore exists inside the walls of HIPE structures as represented as ‘pores’. There are several main routes to produce porous structures including phase inversion and emulsion templating (Gokmen & Du Prez 2012; Wu et al., 2012), porogen incorporation (Ndizeye et al., 2019; Suriyanarayanan et al., 2019; Mohamed and Wilson, 2012), and the direct synthesis of porous organic polymers methods (Zhang et al., 2020a, b). One of the effective strategies leading to hierarchically porous strictures is the emulsion templating technique through the development of high internal phase emulsions (HIPEs), which possess an internal phase volume of above 74 %. This generates a polyhedral shape of the dispersed droplets, whose morphology can be tuned on numerous levels, ranging from overall porosity to pore size to the interconnectivity of pores (Shahbazi et al., 2024; Shahbazi et al., 2022; Hobiger et al., 2021).

Commonly, oil-in-water (O/W) or water-in-oil (W/O) HIPEs can be stabilized using polymeric, surface-active particles, and small molecule surfactants; thus, the type of emulsion dictates whether the HIPE produced is hydrophilic or hydrophobic. To physically stabilize HIPEs, micrometer- or nanometer-scale solid particles are frequently added to HIPE systems, recognized as Pickering-HIPEs (Jiang et al., 2020). While amphiphilic surfactants decrease the interfacial tension of typical emulsions, the solid particles develop a stiff layer that surrounds the dispersed phases, avoiding system coalescence and phase separation (Hunter and Armes, 2020). The size and shape of particles, interparticle interactions, and wetting of particles with liquids control its capacity to stabilize HIPEs. Until now, a diverse range of solid particles such as silica (Ikem et al., 2008; Zheng et al., 2013; Binks et al., 2005), clay (Ashby and Binks, 2000), titania (Ikem et al., 2010; Chen and Zhou, 2015), carbon nanotubes (Briggs et al., 2015; Wang et al., 2012), iron oxide nanoparticles (Lin et al., 2015; Li et al., 2022) and polymer particles (Zhang & Chen, 2009; Zhang et al., 2021) has been effectively utilized to produce colloidally stable HIPEs. Though HIPEs are a basic part of pharmaceutical, cosmetic, and food applications (Bai et al., 2018), either upon processing or in final product shapes, there is a high demand for label-friendly products fabricated from natural and renewable particles (Bai et al., 2017). This stimulates attention to the replacement of non-green nanoparticles with all-natural sustainable ones that serve to stabilize Pickering-HIPEs (Dufresne, 2019). Owing to their multifunctionality, biocompatibility, non-toxicity, and low cost, the application of sub-micrometer-sized solid particles obtained from renewable sources has attracted considerable research interest as a Pickering stabilizer such as gelatin nanoparticles (Tan et al., 2014), cellulose nanoparticles (Pang et al., 2018), globular proteins (Xu et al., 2019, 2020), zein nanoparticles (Dai et al., 2019), and starch nanoparticles (Yan et al., 2019).

There is a growing interest in the use of nano-scale starch particles as starch nanocrystals (SNCs) and starch nanoparticles (SNPs) to develop Pickering-HIPEs because of their outstanding functional properties (Le Corre and Angellier-Coussy, 2014; Campelo et al., 2020; Liu et al., 2021). To yield specific starch nanomaterial types, enzyme debranching, and recrystallization (Kim and Lim, 2009; Sun et al., 2014), precipitation of amorphous starch (Ma et al., 2008), acid hydrolysis (Putaux et al., 2003), and mechanical process (Liu et al., 2009) have been used to obtain nano-scale starches, resulting starch nanomaterials possess different crystallinity, shape, and functionality. The most frequently utilized technique to produce SNCs is acid hydrolysis, which is strongly affected by starch verities. Amylose fraction was reported to jam the pathways for the acid hydrolysis process, leading to increasing particle sizes and decreasing production yield (Lin et al., 2011; Kim et al., 2015). For this reason, starches with low amylose content (e.g., maize and wheat) have been preferably used to prepare SNCs (Le Corre, Bras and Dufresne, 2010). Alternatively, SNPs can be prepared using processing starch in a certain way, including cocrystallization, precipitation, and other approaches with no selective elimination capacity (Haaj et al., 2016), which commonly have a lower crystalline domain and favorable mechanical strength in comparison with SNCs.

Yet, the interfacial properties of neat bio-based nanoparticles are relatively insufficient due to low surface charge and poor hydrophobicity, which can further result in emulsion instability and phase separation (Ortiz et al., 2020). The surface modification of bio-based nanoparticles with a diverse range of organic molecules increases their tendency to transfer across the O/W interface, increasing their colloidal stability of Pickering-HIPEs (Jacob et al., 2018). Fortunately, the presence of a large number of active hydroxyl groups on the surface of bio-based nanoparticles simply reinforces the interfacial properties, therefore improving hydrophobicity, reducing surface polarity, and enhancing emulsion stability (Kedzior et al., 2019). Grafting hydrophobic compounds onto bio-based nanoparticles can effectively improve the wettability of the particles at the O/W interface, decreasing the interfacial tension and regulating the type of emulsion (Kedzior et al., 2019; Wang et al., 2020). Lately, 1,2-butene oxide has been used to fabricate a functionalized hydrophobic nanoparticle with a thermoresponsive behavior (Zheng et al., 2019). To enhance or confer particular functional properties to bio-based nanoparticles and thus expand their application fields, cold plasma has been shown great interest as a non-thermal, facile, low-cost, and clean technique (Yan et al., 2013). This modification method develops free radicals’ initiators onto the polymeric surface, which can lead to the grafting of 1,2-butene oxide onto the biomaterial backbone without the utilization of organic solvent. To induce surface modification, a plasma-induced graft-polymerization can be initially performed followed by a contact of the activated materials with the atmospheric condition or with particular atmospheres, like oxygen, which produces a reactive radical function (such as peroxides) onto the material surface (Dufresne, 2019).

Three-dimensional (3D) printing is an additive manufacturing technique for engineering any 3D data designed with computer-aided design (CAD) programs by adding layers of printable material to a 3D physical part (McMenamin et al., 2014). Compared to classical methods, 3D printing offers a robust capability to fabricate custom-designed and complex 3D structures (Shahbazi et al., 2021). Alternatively, this technique is versatile concerning materials and the versatility of printable material derives from system variation, however, for each particular application such as food printing, the biocompatible materials are still restricted and additional progress of innovative printing materials is still essential (Lee et al., 2017). The utilization of HIPEs in 3D printing is poised to open an innovative viewpoint in the rapid prototyping of personalized 3D objects with enhanced multifunctionality (Sears et al., 2016; Shahbazi and Jäger, 2020). Due to their lightweight and high surface area, 3D printing has recently started to be used to manufacture a highly interconnected macroporous scaffold, allowing them outstanding candidates for a range of applications (Deb et al., 2018). A 3D scaffold with high porosity is tremendously attractive because of its low cost and simple construction approaches, offering a 3D medium for the proliferation, survival, and maturation of cells (An et al., 2015). The functional 3D hierarchically porous structures have been widely developed using utilized biomimetic sacrificial templating compounds, including emulsion templating, colloidal nanomaterials, and gas bubbles. However, the application of these approaches commonly needs a multipart process and organic solvents for crosslinking the porous structures, which are problematic to comprehend a series of hierarchically porous structures, therefore restricting the biocompatibility, targeting feature, adsorption property, and biodegradability with a controllable degradation rate. The term 4D printing is an extension of 3D printing including the presence of a “space-time axis” thanks to the 3D coordinate axis, which has been introduced by Professor Tibbits of Massachusetts Institute of Technology (MIT) (Shahbazi et al., 2023a). Presently, 4D printing is realized using the combination of numerous hydrogel inks concerning a definite formulation and a structural design. In this domain, a shape-transforming feature caused by this combination through the 3D printing methods is a vital research gap, which requires to be addressed in the progress of 4D printing.

To the best of our knowledge, no reports have shown the utilization of modified starch nanomaterials to colloidally stabilized HIPEs served as a printing ink to engineering a thermoresponsive macroporous structure. In this paper, modified SNCs or SNPs with a thermoresponsive feature were initially produced by grafting 1,2-butene oxide onto their structure through a cold plasma grafting method. Afterward, each thermoresponsive starch nanomaterial was individually added to HIPEs to develop a colloidally stable printable ink. In this step, the effect of modified SNCs or SNPs on the physical, rheological, and microstructure of the Pickering-HIPEs was compared. Finally, the prepared Pickering-HIPEs were 3D printed by an extrusion-type printing system to fabricate a thermoresponsive macroporous 3D architecture.

## 2 Methods and materials

### 2.1 Materials

Waxy maize starch (11.2 % w/w moisture content, 0.087 % w/w lipid, 0.21 % w/w protein, >95 % amylopectin, starch damage 1.32 % of starch, and a water holding capacity of 85.2 % w/w) was provided by Sigma-Aldrich, (Steinheim, Germany). Sulfuric acid (98 %) and 1,2-butene oxide were also supplied by Sigma-Aldrich, (Steinheim, Germany) and used as received. Corn oil was purchased from a local market. Nile red and Nile blue '*A*' were provided by Fluka (Buchs, Switzerland). Other solvents and reagents were commercially available and used without further purification unless stated otherwise.

### 2.2 Production and modification of starch nanomaterials

#### 2.2.1 Preparation of SNCs

Native waxy maize starch (73.5 g) was hydrolyzed in a 500 mL aqueous sulfuric acid solution (3.16 M). The suspension was gently stirred by a magnetic stirrer for 1 week at 40 °C to hydrolyze the amorphous fractions of starch (Li et al., 2012). Transmission electron microscopy (TEM) was used to confirm complete hydrolysis through agreement of the final yield of SNCs with previous reports (Campelo et al., 2020). After completion of the hydrolysis process, the SNCs were transferred into centrifuge test tubes, in which centrifugation was performed at 10,000 g for 30 min at ambient temperature through a CR22G high-speed centrifuge (Hitachi Co., Tokyo, Japan). To evaluate the acid-hydrolyzed degree, the first supernatant was kept in a glass container. A consequent washing and centrifugation of the precipitate with distilled water (about 6 consequent wash-centrifugation cycles) was performed until a neutral pH was reached and finally freeze-dried. The production yield of SNCs was determined as the percentage of the residue dry weight at a certain time to an initial dry weight of the starch. Moreover, the percentage of the whole carbohydrate from the initial supernatant at a certain time to the initial dry weight of the starch was considered as hydrolysis degree. Total carbohydrate was analyzed by the phenol-sulfuric acid technique.

#### 2.2.2 Preparation of SNPs

An ultrasound treatment of the starch suspension was conducted to produce SNPs (Agi et al., 2019). Native waxy maize starch suspension was produced by dispersing 2 g waxy maize starch in distilled water to obtain a concentration of 2 g mL^-1^ and stirred at 40 °C for 2 h. Next, it was kept in a glass container and immersed in a water bath kept at a temperature of 5 ± 0.5 °C. Afterward, the suspension was subjected to an ultrasound treatment (VC 155,750, Sonics & Materials, Inc., CT) fitted with a 13 mm diameter probe, processing at a frequency of 20 kHz with a power output of 80 % for 120 min. After completion of the sonication process, the suspension turned transparent from an initial turbid white appearance.

#### 2.2.3 Hydroxybutyl modification of SNCs and SNPs through cold plasma

Hydroxybutylated starch nanomaterials were prepared according to the previous modification procedure with slight modification (Zheng et al., 2019). The starch suspension was prepared by dispersion of SNCs or SNPs (1 g) in deionized water (10 mL) stirred by a magnetic stirrer at 40 °C for 120 min. Each suspension was cooled to ambient temperature followed by a drop-wise introduction of NaOH (10 M) and stirred vigorously for 120 min to make a suspension with a pH of 13. The concentration of the SNC or SNP dispersion was about 25 % (w/w). Afterward, 1,2-butene oxide (10 mL) was incorporated into the suspension and the mixture was stirred through a high-shear rotor-stator device (SilentCrusher 130 M, Heidolph, Germany) operating at 12,000 min^−1^ at 40 °C for 18 h. A dielectric barrier discharge (DBD) plasma device (Nanjing Suman Electronics Company Limited, Nanjing, China), including a DBD-50 reaction cell, voltage regulator, and CTP-2000 K power supplier was used for surface modification of alkalized SNCs or SNPs. The diameter of the quartz circular plate was 100 mm, and the thickness was 1.0 mm. The above solution was carried into the DBD reactor with air as the air source. The cold plasma was employed at a discharge current of 1.5 A, where its discharge voltage was kept at 50 kV with an applied discharge time of 2, 4, and 6 (labeled as SNC-P2, SNC-P4, and SNC-P6 or SNP-P2, SNP-P4, and SNP-P6). The samples containing 1,2-butene oxide but with no plasma treatment were labeled as SNC-P0 or SNP-P0. Next, each treated sample was allowed to stand at room temperature for 120 min and then neutralized with 1 M HCl with vigorous stirring. Dialysis against deionized water was utilized regarding the neutralized suspensions for 36 h by a dialysis bag with a molecular weight cut-off (MW_cut-off_) of 1 kD. A minimum of seven water replacements was accomplished throughout 36 h. The total dilution proportion was about 1:10^10^. Each dialyzed SNC and SNP was lyophilized for 48 h to obtain a white powder.

### 2.3 Characterization of unmodified and etherified starch nanomaterials

#### 2.3.1 Particle size distribution

A temperature-controlled dynamic light scattering (DLS, Malvern, Worcestershire, UK) coupled with a Helium–Neon laser (0.4 mW; 633 nm) was used to detect the particle size distribution of SNP or SNC. The scattered light intensities were measured at 90° to the incident beam. Each sample was diluted in deionized water and analyzed at ambient conditions. The Zetasizer software (version 7.11) was employed to analyze the particle size distribution diagrams, which assessed the ratios of size on the basis of the light intensity detected with the photodetector. The particle size distribution was recorded as the average of three mean measurements.

#### 2.3.2 Transmission electron microscopy (TEM)

The SNCs or SNPs were analyzed using a Hitachi 7700 transmission electron microscope (Hitachi, Tokyo, Japan) at an acceleration voltage of 80 kV. To prepare the sample, a small amount of the diluted SNP or SNC suspensions was placed on a 300-mesh copper grid with holey carbon film. Afterward, excess liquid was removed with filter paper, and the sample was dried under vacuum conditions.

#### 2.3.3 Fourier-transform infrared spectroscopy (FTIR)

A Fourier transform infrared (FTIR) spectrometer (Nicolet iS10, Thermo Fisher Scientific, America) was used to analyze the short-range crystalline properties of native starch, SNCs, and SNPs. To scan with the FTIR spectrometer, the samples were mixed together with potassium bromide (1/100, g/g), ground, and pressed into disks. Then, the spectra were obtained at room temperature in the air, using the FTIR spectrometer in transmittance mode within the wavenumber range of 4000 to 400 cm^−1^. The resolution was set to 4 cm^−1^ and a total of 32 scans were performed.

#### 2.3.4 X-ray photoelectron spectroscopy (XPS)

The dried samples were analyzed using a photoelectron spectrometer (Kratos Axis Ultra DLD, Manchester, UK) to determine the presence of elements such as C, O, S, and Cl. The analysis was performed using Al K*α* X-ray (*hv* = 1486.6 eV) with 5 mA current and 15 kV voltage. The XPSPEAK41 software (version 4.1) was used for data processing after a Shirley-type background correction.

#### 2.3.5 Wide-angle X-ray diffraction (WAXD)

An X-ray diffractometer (XRD, D8 ADVANCE A25, Bruker, Germany) with a target Cu-anode X-ray tube was used to analyze the long-range crystalline properties of different samples. The voltage was set to 40 kV and the current to 40 mA. The diffraction angle (2θ) was scanned at 4–40°, with a scanning step of 0.0196° and a speed of 5.476° min^−1^. The relative crystallinity and average crystallite size of all samples were calculated using GSAS-EXPGUI software.

The relative crystalline degree (*RCD*) was measured by taking the proportion of the amplitude of the crystalline peak at approximately 17° (2θ) ($I_{p}$), to the combined amplitude of the amorphous halo and the crystalline peaks ($I_{t}$) that were measured from the horizontal baseline after drawing a straight line from a diffraction angle of 2θ = 13° to 2θ = 27° Eq. (1):

(1) RCD(%)=(IpIt)×100

#### 2.3.6 Contact angle

The thin films of native starch, unmodified or modified SNC and SNP were prepared on quartz crystal sensors (Biolin Scientific, Gothenburg, Sweden) coated by silicon dioxide. The sensors were first cleaned using dry N_2_ and then placed inside a UV Ozone Cleaner—ProCleaner (BioForce Nanosciences) for 15 min. Thin films of all samples were deposited by spin coating (Chemat Technology Spin-Coater KW-4A, Northridge, CA) at 3000 min^−1^ for 60 s with an acceleration of 2300 s^−1^. The SNC- and SNP-based films were then dried overnight at 45 °C in an oven.

#### 2.3.7 Atomic force microscopy (AFM) observation

To prepare the samples for AFM (SPM-9700, SHIMADZU Corp., Japan), 10 μL of the ethanol solution was added to the samples and then placed onto a mica sheet. The samples were then air-dried and examined using AFM in phase mode to determine average roughness (*R*_a_) and root mean square roughness (*R*_q_).

### 2.4 Preparation and characterization of Pickering-HIPE-based inks

To prepare the Pickering-HIPEs, SNCs or SNPs were suspended in distilled water and stirred overnight to ensure complete hydration. All the Pickering-HIPEs stabilized by SNCs or SNPs were obtained by homogenizing a mixture of 75 % (w/w) corn oil with 25 % (w/w) aqueous dispersion contained SNC or SNP suspensions using a high-shear rotor-stator device (SilentCrusher 130 M, Heidolph, Germany) operating at 12,000 min^−1^ for 2 min. Pickering-HIPEs were then subjected to ultrasound treatment (frequency 20 kHz; 157 amplitude 60 %; power 450 W) for 5 min (with pulse mode durations of 2 s on and 4 s off). The stability and microstructure of these emulsions made freshly or after storage for up to 15 days were compared. The type (O/W, or W/O) of the resultant HIPEs was evaluated by the conventional drop test. If an emulsion could be rapidly dispersed in the aqueous phase (deionized water) but remain agglomerated in the oil phase, it was considered as the O/W type.

#### 2.4.1 Droplet size measurement of Pickering-HIPE-based inks

The Pickering-HIPEs were stirred gently at room temperature to ensure that they were homogeneous. A laser diffraction device (MS 2000, Malvern Instruments Ltd., Worcestershire, UK) was used to measure the droplet sizes and particle size distribution of the HIPEs based on the scattering of a monochromatic beam of laser light (*λ* = 632.8 nm). To determine the size of the droplets, the volume mean diameter was calculated using the expression *d*(_3__*,*__2_) = (∑*n*_i_*d*_i_^3^*/∑n*_i_*d*_i_^2^), where *n* is the number of droplets with diameter *d*_*i*_.

#### 2.4.2 Emulsion stability by vertical laser profiling

The global Turbiscan stability index (*TSI*) parameter is commonly to determine emulsion stability through static multiple light scattering (S-MLS) experiments, accounting for diverse storage processes of emulsion (particle coalescence and settling processes). The stability experiment itself was conducted by vertical laser profiling using a Turbiscan Lab Expert stability analyzer (Formulaction, Toulouse, France) for a duration of 180 min under ambient conditions. The stability of the emulsion was tested using multiple light backscattering of a pulsed near-infrared light (880 nm). The emulsions were placed in a test bottle, with a height of 48 mm. The entire height of the ink was scanned, and the differences in the back-scattering and transmission light (*T*) were detected. The following formula was applied:

(2) T(l,r)=T0exp(2ril)

Here, *r*_*i*_ represents the inner diameter of the sample cell, while *T*_0_ is the transmittance of the continuous phase, which is water:

(3) l(d,φ)=(2d3φQs)

where *d* is the mean diameter of the droplets, *φ* the volume fraction of droplets, and *Q*_s_ is the optical parameter from Mie theory.

The transmittance detector receives light that has passed through the dispersion at an angle of 180° relative to the source, while the backscattering detector receives light scattered backward by the emulsion at an angle of 45°. The *TSI* is used to determine the emulsion stability:

(4) TSI=∑i=1n(xi−xBS)2n−1

where, λ* is the photon transport mean free path, *φ* is the particles' volume fraction, *d* is the particles' mean diameter, and *g* and *Qs* are the optical parameters assumed with the Mie theory. The *χ_i_* is the average backscattering for each minute during the experiment, *χ_BS_* is the average *χ_i_*, and *n* is the number of scans.

#### 2.4.3 Confocal laser scanning microscopy (CLSM) of inks

The interfacial framework and network structure of the continuous phase of the Pickering-HIPEs were visualized using an FV-300 confocal laser scanning microscopy system (CLSM, Olympus, Tokyo, Japan), coupled to an Olympus IX71 inverted microscope and an argon-ion laser. The oil phase was dyed by adding 10 μL of Nile red solution (1 mg mL^−1^ ethanol) to 100 μL of the samples. After being mixed thoroughly using a pipette, 6 μL of the dyed HIPEs was placed on a microscope slide, covered with a glass coverslip (18 mm × 18 mm, Assistant, Sondheim, Germany), and quickly fixed with nail polish to prevent evaporation. The Nile red dye was excited at 488 nm and emitted at 539 nm. All images were taken at 40× magnification and processed with the Olympus Fluoview software (version 2.1, Olympus, Tokyo, Japan).

#### 2.4.4 Static and oscillatory rheological experiments of inks

The rheological properties of the Pickering-HIPEs were analyzed using an AR 2000ex rheometer (TA Instruments, New Castle, DE) with a parallel plate geometry (diameter 40 mm, gap 1 mm). To determine the steady rheological properties, the shear stress (τ) was measured as the shear rate ($\dot{\gamma }$) was increased from 0.1 to 1000 s^−1^.

The oscillatory strain sweep (0.01–100 %, 1 Hz) was conducted to determine the limit of the linear viscoelastic region (*LVR*), while the frequency sweep test (0.1–100 Hz) was carried out within the *LVR* (*γ* = 1 %). All measurements were taken at a temperature of 25 °C.

The time-dependent thixotropic response of Pickering-HIPEs was evaluated by a five-interval thixotropy test (5-ITT) to gather thixotropic data. This test evaluated whether the Pickering-HIPEs rapidly recover upon being sheared at large deformations. The ideal thixotropic structure should show at least 70 % recovery of its peak viscosity in the third interval compared to its initial interval. The 5-ITT detected the viscosity profiles of the samples under alternating low and high shear rates (0.1 and 50 s^−1^, respectively) for 100 s each.

#### 2.4.5 Non-linear rheological response of inks

A Fourier transform (FT) rheology method was applied to quantify viscoelastic non-linearity, which is used in many types of complex fluids including polymer solutions and polymer nanocomposites. In this test, the stress signals were analyzed using FT-rheology, which showed the total non-linear viscoelastic stress. It could be separated into linear viscoelastic stress and odd higher harmonic contribution. The relative third harmonic intensity (*I*_3/1_) was found to be the most intense among the higher harmonics. At small strain amplitude (*γ*_0_), *I*_3/1_ increased as a quadratic function of strain amplitude *γ*_0_. Using the equations suggested by Hyun et al. (2011), a non-linear mechanical coefficient $\left(Q=\frac{I_{j/j}}{\gamma_{0}^{2}}\right)$ and the intrinsic non-linearity $\left(Q_{0}=\lim_{\gamma_{0}\to 0}\frac{I_{j/j}}{\gamma_{0}^{2}}\right)$ (limiting value of *Q* at small shear strain) were calculated based on this scaling relation.

### 2.5 Characterization of 4D-printed objects

#### 2.5.1 The 3D printing of Pickering-HIPEs

The prepared HIPE-based inks were printed through an extrusion-based 3D printer (nScrypt-3D-450**,** nScrypt, Orlando, FL), and connected to a syringe pump (PHD Ultra; Harvard Apparatus Holliston, MA). A special shape of cube or Torres was modeled by the application of computer-aided design software (AutoCAD; Autodesk Inc., San Rafael, CA), and converted to an STL file. Each Pickering-HIPE was then printed in a 3D Torres and cubic through a needle diameter of 1 mm with an extrusion flow speed of 50 mL min^−1^ at an ambient temperature on a special plastic surface. The print paths were provided through the creation of the G-code files to control the XYZ direction instruction of the printer, developed by the open-source CAM software Slic3r (slic3r.org, consulted on September 2022) from the STL file. The printable Pickering-HIPE-based inks were poured into a stainless-steel cartridge (10 mL) and stirred with a Vortex mixer (Fisher Scientific, Ontario, Canada) for 10 min to remove the air bubbles from the ink. Finally, each Pickering-HIPE was processed with the 3D printer using a needle diameter of 1 mm with the extrusion flow speed of 30 mL min^−1^ at 30 °C on a special plastic surface. To evaluate the thermoresponsive of the printed Pickering-HIPEs, the 4D printed objects were alternately washed with absolute ethanol and deionized water to remove the internal phase, followed by a freeze-drying process (Martin Christ, Alpha 2–4 LD Plus, Osterode am Harz, Germany) to yield a freeze-dried oil-free 3D structure.

#### 2.5.2 Morphological evaluations of 3D printed structures

The FV-300 CLSM (Olympus, Tokyo, Japan) was used to assess the microstructural observation of free-oil freeze-dried 3D-printed objects. Moreover, the morphological structure of the 3D ‘grid’ was captured through a field-emission scanning electron microscope (FE-SEM, S-4700, Hitachi, Japan) to produce high-resolution images with a high depth of field. Initially, each 3D construct was cut into a precise size of (15 × 15 × 15) mm^3^. Next, the sectioned 3D printed samples were mounted on a Peltier-cooled stage with a temperature of −10 °C to avoid thermal damage. The nitrous oxide was utilized as an imaging gas that offered a pressure of 50.7 Pa. The microstructures of each 3D construct were obtained through a solid-state backscatter detector via an accelerating voltage of 20 kV.

### 2.6 Demonstration of stimuli-response of printed Pickering-HIPEs

To detect the thermoresponsive behavior of 3D printed structures, the resulting 3D printed constructs were alternately washed with absolute ethanol and deionized water to remove the internal phase, followed by freeze-drying to explore the thermoresponsive properties. A UV–Vis spectrophotometer (UV-2600, Shimadzu, Japan) equipped with a temperature controller was used to evaluate the lower critical solution temperature (LCST)-type phase transition of 3D printed structures at a fixed wavelength of *λ* = 650 nm. Regarding this experiment, typically the samples (1.5 mL) were dispersed in dimethylsulfoxide (DMSO) and filtered through a membrane filter with a pore size of 0.22 μm. About, 1 mL from each dispersion was taken in 1 mL of quartz cuvette and the transmittance (%) was recorded in the temperature interval of 20–60 °C with a heating/cooling rate of 1 °C min^−1^.

### 2.7 Hydrodynamic radius detected by DLS

The hydrodynamic radius (*R*_h_) was measured by Malvern Autosizer spectrometer (4700 DLS Malvern, Worcestershire, UK) at 25 °C and 90° scattering angle. The *R*_h_ was measured by cumulant analysis.

### 2.8 Statistical analysis

All instrumental experiments were carried out as triplicate determinations and the mean and standard deviation of the data were reported. Analysis of variance (ANOVA) was utilized the determine the main effects of the examined independent factors and their interactions with the instrumental data. Duncan's multiple range test was applied to separate means of data when significant differences (*p* < 0.05) were observed.

## 3 Results and discussion

### 3.1 Characterization of modified SNCs or SNPs

#### 3.1.1 Effect of hydroxybutyl modifications on the morphology of SNCs and SNPs

**Fig. 1:**
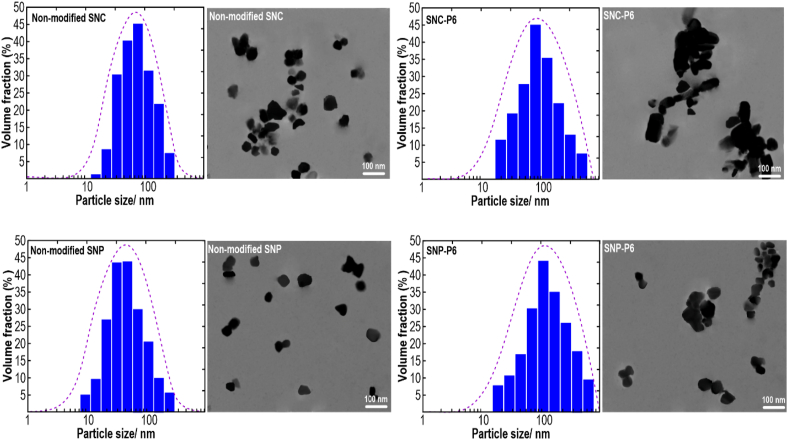
The particle size distribution of both SNCs and SNPs as affected by hydroxybutyl modification determined by DLS and related TEM images.

The chemical modification of starch nanomaterials notably affects their functionalities such as crystallinity (Pérez and Bertoft, 2010), emulsification (Agama-Acevedo and Bello-Perez, 2017), water binding capacity (Singh et al., 2003), and formation of starch polymer network (Hoover, 2001). Native waxy starch granules offered a spherical shape with a diameter of about 8–94 μm (data not shown), which was in accordance with the results of Sun et al. (2014). After producing unmodified SNCs or SNPs, both starch nanomaterials were obviously in the nanoscale range with a mean particle size of below 150 nm, which also showed a monomodal size distribution (Fig. 1). Compared to unmodified samples, modified SNPs or SNCs presented bigger sizes with some agglomerated particles, which is likely due to hydroxybutyl modification of SNCs and SNPs.

The morphology of both starch nanomaterials was evaluated by TEM observation (Fig. 1). The unmodified SNCs presented a platelet-like geometry with a well-defined parallelized shape and the presence of some particle aggregates, whose size ranged from 25 to 90 nm. The unmodified SNPs possessed a spherical form in addition to the attendance of some local aggregate, whose size ranged from about 20 to 80 nm. Inversely, both modified SNCs and SNPs presented a high level of particle size larger than 200 nm, which could be the result of the strong tendency of particles to self-aggregate during hydroxybutyl modification. The self-aggregation of SNCs and SNPs was favored by their platelet and spherical morphologies, respectively, and the presence of surface hydroxybutyl groups favoring interaction through ester and hydrogen bonding.

#### 3.1.2 FTIR experiment

**Fig. 2:**
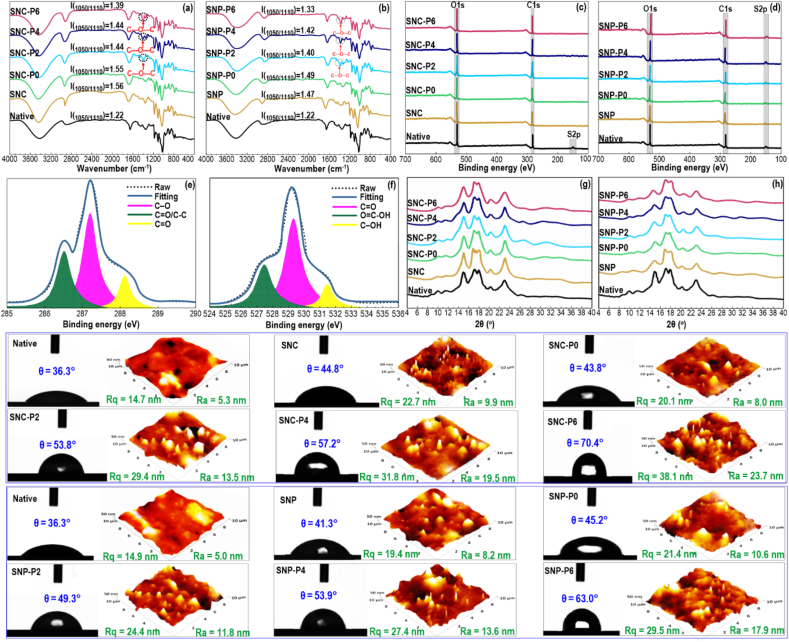
(Top): Characterization of native waxy maize starch, unmodified or modified SNCs and SNPs: (a,b) FTIR, (c,d) XPS, (e,f) Zoomed XPS, (g,h) XRD. (Bottom): Contact angle measurement and related AFM images of native starch and modified SNCs or SNPs.

The FTIR experiment was used to verify starch nanomaterials etherification and also to evaluate short-crystalline features of native waxy maize starch and its resultant SNC or SNP products (Fig. 2a and b). The IR spectrum of native waxy maize starch showed several obvious pronounced peaks ranging from 4000 to 400 cm^−1^. The absorption band at 3558 cm^– 1^ is related to the stretching of the –OH group in glucose units. The intense peak was also observed at 2881 cm^−1^ resulting from an asymmetric C–H stretching vibration in the –CH_2_ group. Furthermore, a typical band at 1622 cm^−1^ is assigned to the bending vibration of O–H. This represents the characteristic peak of tightly bound water existing in starch, which has been established in the literature (Ma et al., 2008; Yu et al., 2010). The symmetrical bending vibration of the C–H within the –CH_2_ group was reflected in the peak of 1410–1525 cm^−1^. The characteristic band at 1110 cm^−1^ signifies a stretching vibration of the C–C skeleton and usually acts as an internal correction standard of short-range order structure. The absorption peak at 1050 cm^−1^ corresponds to the short-crystalline structure, while the typical peak at 1020 cm^−1^ represents the amorphous structure of starch (Warren et al., 2016). Therefore, the intensity of 1050/1110 cm^−1^ (*I*_1050/1110_) and 1020/1110 cm^−1^ (*I*_1020/1110_) commonly shows the short-crystalline structure and degree of amorphous, respectively, where a double helix structure is developed between amylopectin and/or amylose short chains (Hu et al., 2023).

Compared to native waxy maize starch, the IR characteristic peaks of non-etherified SNCs or SNPs were found not to be changed, nor did an emergence of a new peak. In this case, the chemical structure of waxy maize starch did not alter after the acid hydrolysis process (regarding SNCs) and sonication treatment (SNPs), particularly for the bands at 1140 and 870 cm^−1^, which were related to S=O and C–O–S stretching vibrations, respectively, thus confirming the formation of sulfate ester (Wei et al., 2014). Typically, the band at 800–1200 cm^−1^ in the FTIR spectra was selected and deconvoluted for analyzing the short-crystalline structure of starch. The results were measured and included in Table 1. As given in Fig. 2 and Table 1, the *I*_(1050/1110)_ value of the SNCs or SNPs was greater compared to native waxy maize starch, signifying that more crystalline segments were produced from native granules. This phenomenon may verify that the chemical hydrolysis or sonication treatments were proper to develop SNCs or SNPs with a short-crystalline structure. Compared to native waxy maize starch, the value of *I*(_1020/1110_) of SNCs or SNPs was also higher, which may elucidate why the more amorphous segment was developed from native granules destroyed by acid hydrolysis or sonication. The *I*(_1020/1110_) of SNCs or SNPs samples increased with increasing hydrolysis time, which is likely associated with the decomposition of native granules by sulfuric acid or sonication, which efficiently eliminates some amorphous areas of starch and retains its crystalline fractions. It was also possible that the uncoupling of a single double helix from the amylopectin backbone produced from acid hydrolysis eliminated the space restriction, making the double helix reordered to a more crystal structure (Vermeylen et al., 2004).

**Table 1:** Summary of obtained results for *I* (_1050/1110_) and *I* (_1020/1110_) parameters detected by FTIR, contact angle, and AFM parameters for native starch, unmodified, or modified SNCs and SNPs.

Sample	*I*_*(*1050/1110)_	*I*_*(*1020/1110)_	Contact angle (^o^)	AFM parameters	
				*R*_*q*_ (nm)	*R*_*a*_ (nm)
Native	1.22 ± 0.02^a^	1.31 ± 0.01^a^	36.3 ± 0.7^a^	14.7 ± 0.2^a^	5.3 ± 0.1^a^
SNC	1.56 ± 0.02^h^	1.61 ± 0.04^cd^	44.8 ± 0.7^cd^	22.7 ± 0.7^d^	9.9 ± 0.3^c^
SNC-P0	1.55 ± 0.02^h^	1.60 ± 0.04^c^	43.8 ± 0.7^c^	20.1 ± 0.6^c^	8.0 ± 0.1^b^
SNC-P2	1.44 ± 0.02^e^	1.63 ± 0.05^d^	53.8 ± 0.6^f^	29.4 ± 0.4^g^	13.5 ± 0.2^e^
SNC-P4	1.44 ± 0.01^e^	1.67 ± 0.01^f^	57.2 ± 0.6^g^	31.8 ± 0.7^h^	19.5 ± 0.2^g^
SNC-P6	1.39 ± 0.02^c^	1.67 ± 0.01^f^	70.4 ± 0.9^i^	38.1 ± 0.7^i^	23.7 ± 0.3^h^
SNP	1.47 ± 0.01^f^	1.58 ± 0.01^b^	41.3 ± 0.6^b^	19.4 ± 0.4^b^	8.2 ± 0.2^b^
SNP-P0	1.49 ± 0.01^g^	1.64 ± 0.02^e^	45.2 ± 0.5^d^	21.4 ± 0.3^d^	10.6 ± 0.4^c^
SNP-P2	1.40 ± 0.02^c^	1.69 ± 0.01^g^	49.3 ± 0.7^e^	24.4 ± 0.5^e^	11.8 ± 0.3^d^
SNP-P4	1.42 ± 0.01^d^	1.73 ± 0.02^h^	53.9 ± 0.6^f^	27.4 ± 0.7^f^	13.6 ± 0.2^e^
SNP-P6	1.33 ± 0.02^b^	1.78 ± 0.02^i^	63.0 ± 0.9^h^	29.5 ± 0.7^g^	17.9 ± 0.3^f^

The means in each column with various letters (a–i) are significantly different (*p* < 0.05) according to Duncan's test.

Compared to non-etherified SNCs or SNPs, the SNC-P0 or SNP-P0 (samples containing 1,2-butene oxide but without plasma treatment) presented a comparable IR spectrum with no emergence of new characteristic bands from 4000 to 1400 cm^−1^, showing similar *I*_(1050/1110)_ value (Table 1). However, a new absorption peak near 1372 cm^−1^ emerged on the IR spectrum of all hydroxybutyl SNCs or SNPs, which was caused by the vibration of C–O–C, verifying starch etherification. Furthermore, as plasma discharge time increased, the intensity of this newly developed absorption peak was slightly increased, showing that hydroxybutyl was successfully grafted onto starches. As shown in Table 1, the *I*_(1050/1110)_ value of SNC or SNP (*i.e.*, unmodified SNC or SNP) was greater compared to native waxy maize starch, signifying that more crystalline segments were produced during sulfuric acid or sonication treatments. In this case, SNC offered a higher *I*_(1050/1110)_ value than SNP, suggesting the attendance of a more short-range order structure. After the addition of 1,2-butene oxide upon alkaline condition (SNC–P0 or SNP-P0), its *I*_(1050/1110)_ value was unchanged as 1,2-butene oxide principally attacked the amorphous areas of starch backbone, therefore showing negligible impact on the starch crystallinity (Kim et al., 2011). However, the *I*_(1050/1110)_ value of native waxy maize starch was reduced after hydroxybutyl grafting of SNCs and SNPs using cold plasma. Though the hydroxybutylation modification can occur in the amorphous region, the reaction can still diminish the rigid structure of the crystalline area at low levels (Chen et al., 2021).

#### 3.1.3 XPS assay

The hydroxybutyl modification of SNCs and SNPs was further analyzed by accomplishing the XPS assay and the corresponding XPS spectra are shown in Fig. 2c and d. As can be seen, all XPS survey spectra exhibited two distinct peaks located at 287.5 eV and 529.5 eV, attributing to the characteristic peaks of C1s and O1s respectively. This proposes that native waxy maize starch, SNCs, and SNPs were mostly composed of C and O elements (Xiong et al., 2008). Regarding native starch, the small peak was located at 167 eV, which is assigned to the typical peak of S2p. This band is related to the attendance of sulfur-comprising amino acids in the starch sample. In this case, the XPS spectrum of unmodified SNP did not change in comparison with native waxy maize starch. In contrast, the S2p peak on the surface of unmodified SNC disappeared, which is likely a result of the formation of sulfuric acid esters on the surface of starch during sulfuric acid hydrolysis (Fig. 2c and d). The grafting of 1,2-butene oxide onto the SNCs and SNPs through cold plasma led to no change in the typical peak of S2p of these samples.

The extent of detected elements on the surface of each sample, as well as the ratio of oxygen to carbon (O/C), are presented in Table 2. The O/C of native waxy maize starch was 0.52, which denotes a lower ratio compared to the theoretical value of 0.83. This reveals that native waxy maize starch contained high-carbon impurities, mostly proteins and fats (free fatty acids) (Rindlav-Westling and Gatenholm, 2003; Angellier et al., 2005). After sulfuric acid hydrolysis or sonication, the O/C value decreased, indicating that the C content on the starch surface increased. Table 2 shows that the obtained O/C ratio for all hydroxybutyl SNCs or SNPs was decreased. Similarly, there is a considerable rise of the C–O peak in the C1s high-resolution spectra for chemical analysis of modified SNCs or SNPs (Fig. 2c and d). This proposes that the hydroxybutyl chain was successfully grafted. The obtained data for the modified SNCs or SNPs was not surprising because carbon contents were increased after the grafting process, whereas oxygen contents were decreased compared to native waxy maize starch. The O/C value of SNC-P6 was the lowest among all samples, which is likely due to the effective grafting of 1,2-butene oxide onto SNCs or SNPs by cold plasma. This result is in accordance with the FTIR analysis.

**Table 2:** Elemental surface composition (atomic %) and oxygen to carbon ratio in different samples.

Samples	O	C	S	N	O/C
Native	32.76	63.00	0.26	0.62	0.52
SNC	28.79	70.23	0.21	–	0.41
SNC-P0	28.69	73.56	0.28	–	0.39
SNC-P2	30.22	77.50	0.26	0.62	0.39
SNC-P4	27.61	78.89	0.21	–	0.35
SNC-P6	27.76	79.31	0.28	–	0.35
SNP	30.22	65.70	0.26	0.62	0.46
SNP-P0	29.95	69.66	0.21	–	0.43
SNP-P2	28.98	72.46	0.28	–	0.40
SNP-P4	30.57	76.43	0.21	–	0.40
SNP-P6	28.83	77.91	0.28	–	0.37

Fig. 2e and f presents a typical high-resolution XPS spectra of O1s and C1s (due to a higher involvement in the grafting process). After deconvolution through a least-square peak assay software (XPSPEAK version 4.1), the C1s spectra, showing the hydrophobic character of the surface, were resolved into three peaks at binding energy of 285.5, 287.5, and 289.5 eV, indicating the presence C–O, C–C/C=O, and C=O chemical bonds in the molecular structure of SNC-P6 as an instance (all samples offered a similar XPS resolved peak). For this reason, the hydroxybutyl modification could develop strong interactions between the nonpolar group, increasing the hydrophobicity of modified SNCs or SNPs. Meanwhile, the typical O1s high-resolution spectrum is also divided into three peaks located at various binding energies, such as C–OH, O=C–OH, and C=O, which the O1s peak was also decreased after the etherification process (Fig. 2c and d). In conclusion, the above analyses and investigations of the XPS spectra meaningfully established the effective development of grafted hydroxybutyl SNCs or SNPs (Moreira et al., 2017).

#### 3.1.4 XRD experiment

The hierarchical organizations of starch in natural materials accompanied by their semi-crystalline structure have been an important factor in the production of nanocrystals and nanoparticles through controlled physical and chemical treatments (Le Corre, Bras and Dufresne, 2010). The XRD analysis was performed to evaluate the long-range crystalline structure of the produced SNCs or SNPs and their etherified products. The native waxy maize starch showed a characteristic reflection at 2θ = 15.1° and 2θ = 23.0° with a dual adjacent shoulder peak at 2θ = 17.4° and 2θ = 18.1°, presenting a semi-crystalline structure (Fig. 2g and h). This offers a typical A-type diffraction pattern with a relative crystallinity of 34.9 %. A minor V-type diffraction peak was also detected at 2θ = 20.2°, which is assigned to a complex developed of the amylose-lipids single helices. This result agrees well with the previous reports on crystal structures of native waxy maize starch (Shahbazi et al., 2022). The diffraction peak of non-etherified SNCs showed a similar diffractogram compared to that of the native sample, still an A-type pattern (Fig. 2g), which is in accordance with previous publication (Li et al., 2023). However, the intensity of characteristic peaks notably increased after acid hydrolysis, in which the relative crystallinity of non-etherified SNCs was increased to 55.6 %. Several hypothetical mechanisms have been proposed for increasing the relative crystallinity of starch after acid hydrolysis. A main phenomenon could be the breakdown of amylose passing through the amorphous areas, which might reorganize the newly free chain ends into a more crystalline region (Kainuma and French, 1971). Moreover, the retrogradation of free amylose after acid hydrolysis can increase crystallinity due to the formation of the double helices, which this rearranged structure resists against acid hydrolysis (Atichokudomchai et al., 2004). As presented in Fig. 2g, the sonication process did not also alter the crystalline type of the native sample, in which the produced non-etherified SNPs remained A-type. However, a phenomenon should be emphasized. Compared to the native sample, the typical reflections at 2θ = 15.1°, 2θ = 17.4°, and 2θ = 18.1° gradually smoothened after the sonication process. This reveals that the crystalline structure of native waxy maize starch was disrupted after the sonication process. It was believed that the ultrasonic treatment strongly destroyed the crystalline domains of starch. This results in the produced SNP losing its molecular order in the crystalline lamellae organization of starch chains. It should be noted that in both non-etherified SNCs or SNPs, the V-diffraction peak at 2θ = 20.2° does not seem to be affected by acid hydrolysis and ultrasonication treatment.

As shown in Fig. 2g and h, the diffraction peaks of SNC-P0 or SNP-P0 were found not to be changed, which still remained an A-type crystalline pattern, matching those of native and non-etherified SNCs/SNPs. Similarly, the diffraction pattern of native waxy maize starch was unaffected after grafting by 1,2-butene oxide under alkaline conditions. As mentioned above, 1,2-butylene oxide mainly attacked the amorphous areas of starch therefore having very little impact on its crystallinity (Kim et al., 2011). However, the initial relative crystallinity of SNCs/SNPs was slightly reduced by hydroxybutylation. Although the hydroxybutyl grafting happened in the amorphous region, the process can still destroy the rigid structure of the crystalline segments at low levels.

#### 3.1.5 Contact angle and AFM

Contact angle measurement is an indicator of the surface wettability of a polymer-based film by a liquid, which is normally applied to show its hydrophobic and hydrophilic properties. Fig. 2, Bottom illustrates the changes in the contact angle of waxy maize starch-based film (which was produced by KW-4A spin coater as mentioned in section 2.2.4) as affected by hydroxybutyl modification. The native waxy maize starch film presented a low water contact angle of θ = 36.3° (Table 1). This could be ascribed to a high level of polar groups in the starch structure, which endows a highly hydrophilic and wettable character. Compared to native waxy maize starch, the contact angle of non-etherified SNCs or SNPs was found to be increased. By comparing the AFM images, the *R*_*q*_ and *R*_*a*_ of these samples were increased (Table 1), which contributed to an improvement in their surface roughness. It has been demonstrated that micrometer-size surface roughness strongly affects the surface hydrophobicity of polymeric films (Miller et al., 1996). As another explanation, the XRD measurements showed that the relative crystallinity of non-etherified SNCs or SNPs was increased. It has been reported that there is a linear relationship between the contact angle and crystallinity (Mohammadi et al., 2023). Similarly, after plasma-induced grafting of 1,2-butene oxide upon alkaline conditions (etherified SNCs or SNPs), the surface hydrophobicity was further enhanced and contact angle values were progressively increased with increasing plasma discharge time. This demonstrates the grafting of more 1,2-butene oxide on modified SNCs or SNPs. As XPS results revealed, the hydroxybutyl modification developed effective grafting of the nonpolar group onto the starch structure, increasing the hydrophobicity of modified SNCs or SNPs. In accordance with contact angle results, the *R*_*q*_ and *R*_*a*_ of etherified SNCs or SNPs markedly increased (Table 1), whose 3D AFM images presented an uneven surface topology with many pits and hills on their surface. This provides more rigidity for these samples. The surface irregularity is likely described by the presence of some crystalline fragments of starch. As declared in section 3.1.4, the etherification process can still diminish the firm structure of the crystalline parts even at a low level; thus, some free crystalline segments may be present on the surface of modified SNC- or SNP-based films.

### 3.2 Characterization of Pickering-HIPE-based inks

#### 3.2.1 Droplet size, colloidal/gravitational stability, and microstructure of Pickering-HIPEs

**Fig. 3:**
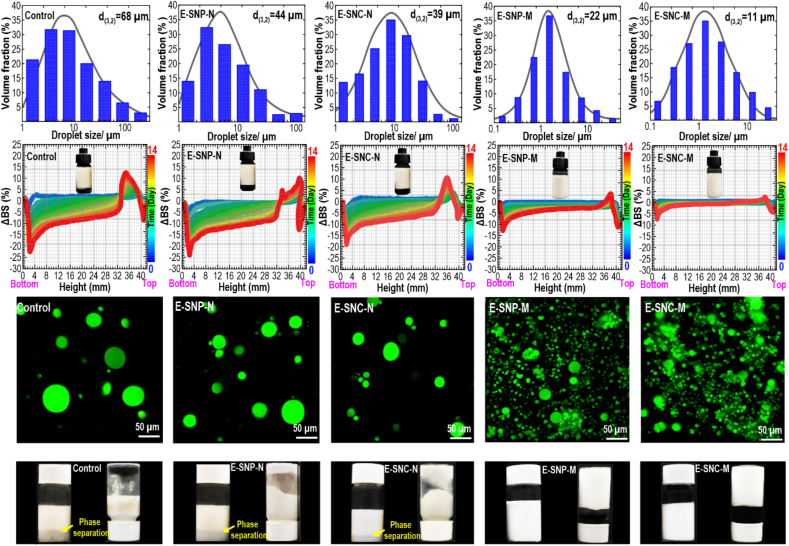
(Row *i*): *PSD*, (Row *ii*): *BS* intensity profiles, and (Row *iii*): Confocal images (the top layers collected for each sample) of different Pickering-HIPEs. (Row *iv*): Visual observation of HIPEs (96 h after preparation).

SNC-P6 and SNP-P6 were chosen as the optimum particulate-type emulsifier to evaluate their ability to stabilize the HIPEs due to excellent long-range crystalline structure and more hydrophobic character. This gives a certain level of hydrophobicity, which is favorable for the colloidal stability of HIPEs. Fig. 3 (Row *i*) shows the particle size distribution (*PSD*) of Pickering-HIPEs. As expected, the *PSD* of the control HIPE (with no particulate emulsifier) presented a wide range of droplet sizes. Similarly, the *PSD* of the HIPEs prepared by unmodified SNCs (E–SNC–N) or SNPs (E-SNP-N) also presented an important coalescence with a big size of droplets, which may be due to a lower surface coverage of unmodified starch nanomaterials on the oil droplets. Inversely, the droplet sizes of HIPEs produced by modified SNCs (E–SNC–M) or SNPs (E-SNP-M) were notably small, where *PSD* shifted to the smaller droplet sizes. This effect may be due to the occurrence of an extensive flocculation, exhibiting a high level of colloidal stability (Ettelaie and Akinshina, 2014). Thus, this phenomenon may highlight that most of modified starch nanomaterials effectively adsorbed at the oil-water interfaces. It is then concluded that both etherified starch nanomaterials were efficient to inhibit the droplet coalescence than the unmodified starch nanomaterials. Compared to E-SNP-M, E–SNC–M showed a smaller droplet size. In this case, the modified SNCs presented a high crystalline structure (Fig. 2g) with more hydrophobic character (Fig. 2, Bottom), then it possessed more efficient role to decrease droplet size, preventing the occurrence of coalescence.

The static multiple light scattering (S-MLS) experiment was used to evaluate the gravitational stability of Pickering-HIPEs by plotting backscattering (*BS*) intensity profiles during 14 days of storage (Fig. 3, Row *ii*). In this case, the Y-axis shows the variation percent of the backscattering light intensity (*ΔBS*) compared to an initial state, whereas the X-axis displays the height of the HIPEs (Shahbazi et al., 2021). Regarding control sample, there was a reduction of *ΔBS* at the bottom of the tube with increasing storage time because of an upward migration of droplets (clarification). Simultaneously, the *ΔBS* in the upper part was increased due to increasing the concentration of droplets in the upper layer (creaming). A similar change in the *ΔBS* was detected concerning E–SNC–N and E-SNP-N, where the peak widths at the left or right side of the *BS* profile showed a high level of *ΔBS*. This denotes the occurrence of clarification and creaming phenomena in these systems. The poor gravitational stability of E–SNC–N and E-SNP-N can be assigned to the high hydrophilic character (Fig. 2 Bottom) of their relevant unmodified emulsifiers, *i.e.*, non-etherified SNCs or SNPs. The low level of hydrophobic property of particulate emulsifiers reduces their tendency for adsorption at the O/W interfaces, which leads to poor colloidal stability. However, the migration rate of droplets in E-SNP-M and E–SNC–M was considerably reduced due to the improved colloidal properties of etherified SNCs or SNPs. Compared to E-SNP-M, the *ΔBS* profiles of E–SNC–M remained rather unchanged at the bottom and top of the testing tube with time. The surface-active SNCs or SNPs can rationally develop aggregated networks with the formation of small droplet sizes (Shahbazi et al., 2022b).

To reveal the fundamental mechanism for the outstanding emulsification process of the etherified SNCs or SNPs, the flocculation of droplets in the Pickering-HIPEs was evaluated through a CLSM observation (Fig. 3, Row *iii*). Similar to the big droplet size occurred in control HIPE, E–SNC–N and E-SNP-N had a comparable large droplet size due to the oil coalescence. However, the existence of some flocculated droplets was also observed in E–SNC–N, which might be attributed to the partial aggregation of droplets. The large oil droplets resulting from coalescence verify that the interfacial layer of non-etherified SNCs or SNPs could not efficiently protect the droplets against phase separation. Evidently, the droplets in E-SNP-M and E–SNC–M showed much smaller sizes with a high level of droplet aggregation, which were tightly associated, and a portion of flocculated and clustered droplets could be developed. The flocculation degree is valuable for the physical stabilization of Pickering-HIPEs, where the adsorbed particles are shared between the pairs of adjacent droplets (bridging flocculation) (Ding et al., 2017). The CLSM images also agree well with the droplet size distributions of the HIPEs, with flocculation being apparent in the emulsion stabilized by etherified SNCs or SNPs. It is worth mentioning that If droplets in this system remain physically stable against coalescence, then the resulting network formed by the flocculated oil droplets can offer strong viscoelastic property (Shahbazi et al., 2023b).

To further quantify the colloidal stability of Pickering-HIPEs, their visual appearance against creaming was assessed. Fig. 3, Row *iv* shows the creaming behaviors of the Pickering-HIPEs after one month of storage. The control HIPE formed a creamed layer at the top of the container, coexisting with an aqueous phase subnatant. As can be seen in Fig. 3 (Row *iv*), the non-etherified SNCs and SNPs were also unable to stabilize HIPEs, and the oil droplets were prone to coalesce in E–SNC–N and E-SNP-N. This can be associated with the lack of sufficient surface coverage of these particulate emulsifiers on the oil droplets, which is likely due to a low level of surface activity as they showed a hydrophilic nature (Fig. 2, Bottom). Inversely, the etherified SNCs or SNPs inhibited the phase separation, where E–SNC–M and E-SNP-M samples were physically stable against creaming after one month of storage. Moreover, these systems showed a gel-like structure and could remain their shape even though the tubes were inverted (Fig. 3, Row *iv*). It is perhaps relevant here to recall that the flocculated network structure formed a 3D gel-like structure, which develops a network of droplets held together by etherified SNCs or SNPs on their surface. This is likely related to their good emulsifying features resulting from higher surface hydrophobicity (Fig. 2, Bottom).

#### 3.2.2 Viscoelastic properties, flow behavior, and thixotropic features of Pickering-HIPEs

**Fig. 4:**
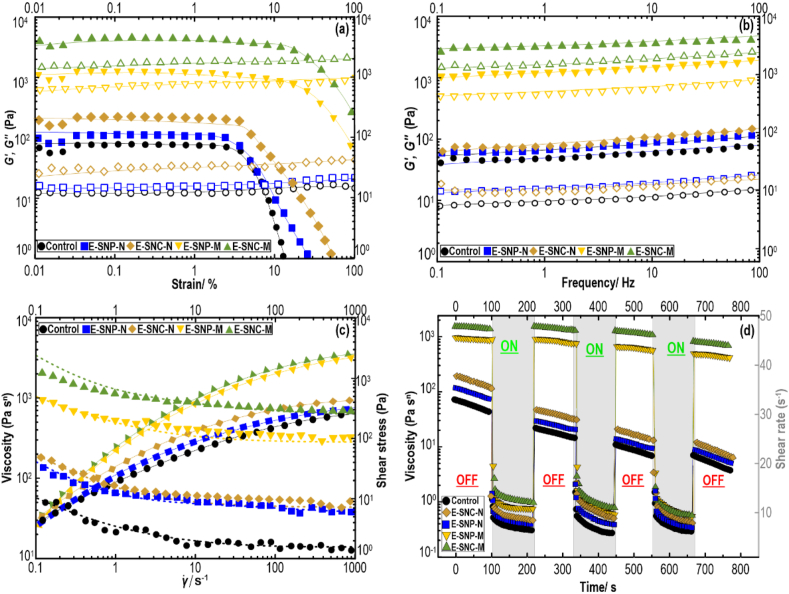
Storage (*G′*) and loss (*G″*) moduli as a function of (a): strain and (b): frequency, where *G′* is solid symbols and *G″* is open symbols; (c): Changes in viscosity and shear stress as a function of shear rate; and (d): viscosity dependence on applied time and deformation rate measured for different Pickering-HIPE-based inks.

The evaluation of dynamic rheological properties of printing inks can endow valuable insight into their viscoelastic behavior, which is directly related to the printability and mechanical strength of resulting printed objects (Shahbazi et al., 2022a). The storage (*G′*) and loss (*G″*) moduli of Pickering-HIPE-based inks as a function of strain are illustrated in Fig. 4a. Regardless of sample type, *G′* (*γ*) was generally higher than *G″* (*γ*) at the lower strain, which denotes the HIPEs possessed a quasi-solid response in this strain range. With increasing the magnitude of strain, *G′* (*γ*) values importantly reduced and finally crossed over with those of *G″* (*γ*). This reveals a change from a more quasi-elastic property to a more viscous liquid-like behavior (Watanabe, 1999). In this situation, the HIPEs cannot endure against the applied strain, whose rheological properties progressively shift from elasticity to viscosity. The control, E–SNC–N, and E-SNP-N inks presented somewhat similar viscoelasticity in the entire strain sweep range, yet the E–SNC–N showed a higher *G′* (*γ*) value. Alternatively, there was a much greater increase in *G*′ (*γ*) values regarding E-SNP-M and E–SNC–M, showing the presence of a more structured gel-like network than other inks. This improved viscoelastic behavior is representative of highly concentrated or flocculated systems. The flocculated emulsion (together with a *pseudoplastic* behavior) stands a chance of 3D printing well, which can produce a favorable 3D structure with high shape-fidelity (Shahbazi et al., 2022b).

Additionally, the length of *LVR*, where the *G′*_*LVR*_ and *G″*_*LVR*_ remain independent of the applied strain, for different Pickering-HIPE-based inks was also evaluated. The *LVR* length reflects the magnitude of the mechanical strength of the system, where stiffer structured HIPEs can remain within the *LVR* over a higher strain level compared to a weak gel. As Fig. 4a depicts, control, E–SNC–N, and E-SNP-N showed a short *LVR* length, presenting a low critical strain (the value at which the *G′*_*LVR*_ deviated by more than 5 % from the prior magnitude in the strain range of 0.1–10 %) of <10 %. Contrarily, the critical strain values of E-SNP-M and E–SNC–M were detected to be >10 %. Thus, these inks could rationally develop a colloidally stable network due to the presence of flocculation (Fig. 3, Row *iii*), leading to a less restricted *LVR* with more structured systems under nondestructive conditions.

Fig. 4b shows the oscillatory frequency experiment results of Pickering-HIPE-based inks. Throughout the entire period of the frequency sweep, the *G′* (*ω*) of all samples was greater than the G″ (*ω*), exhibiting predominantly elastic-dominated behavior. The viscoelastic moduli of all Pickering-HIPEs also revealed a slightly frequency-dependent manner, but no crossover was detected for the *G′* (*ω*) and G″ (*ω*) parameters. Irrespective of sample type, the evolutions of *G′* (*ω*) and *G″* (*ω*) of all the emulsions were quite comparable to the results of strain sweeps. In this case, the magnitude of viscoelastic moduli regarding E–SNC–N and E-SNP-N HIPEs was quite low, which is likely due to the lack of presence of flocculated droplets in these systems. On the contrary, the viscoelastic moduli showed a higher value in E-SNP-M and E–SNC–M, which was in complete accordance with the strain sweep measurements in *LVR*. The modified SNCs and SNPs in these HIPEs led to the formation of a compact arrangement of smaller droplets, which possesses more efficient flocculation and higher *G′* (*ω*). The *G′* (*ω*) parameter shows a sign of the system rigidity; then, the inks with a greater storage modulus offer a more desirable mechanical strength and may aid to form a well-defined geometry during 3D printing process.

The emulsions with a high degree of *pseudoplasticity* and viscosity are also required for the 3D printing process, where the printable systems must be simply squeezed out through the nozzle tip, yet should keep their shape during extrusion force in the 3D printer. Fig. 4c illustrates the flow curves of Pickering-HIPE inks, which was provided by plotting the recorded shear stress or apparent viscosity as a function of the applied shear rate. With increasing shear rate, shear stress was increased non-linearly, which denotes that all Pickering-HIPEs showed a shear-thinning property. Compared to control, the E-SNP-M and E–SNC–M presented a much more appreciable increase in the shear stress with shear rate. This could be a sign that the high level of aggregated/flocculated droplets breaks into smaller clusters under increasing shear rate, causing shear-thinning behavior. The rheological data also showed that E–SNC–M had a higher apparent viscosity compared to E-SNP-M. The flocculation phenomena of the entangling adjacent droplets improved the shear-thinning feature and induced higher apparent viscosity than that of the non-flocculated system. This is important for 3D printing, as the flocculated droplets preserve some of the continuous phases inside their structures, which results in improved printing quality.

To gain insights into the structural recovery property of Pickering-HIPEs, a time-dependent thixotropic response was carried out. Each Pickering-HIPE-based ink was exposed to a five-interval time test (5-ITT), where the changes in samples’ viscosity were evaluated as a function of time under changing constant shear rates (0.1, 50, 0.1, 50, and 0.1 s^−1^). The viscosity plots show (Fig. 4d) that all the Pickering-HIPEs revealed an important drop in viscosity over time in the first and second intervals. This suggests that the response of system relates not only to an induced shear rate but also to the time duration for which the samples have been sheared. Throughout the time-dependent thixotropic evaluation, it could be observed that the viscosity values were slightly reduced with time at the low shear of 0.1 s^−1^; in the meantime, the shear sensitivity became evident at a high-shear interval of 50 s^−1^. Moreover, Pickering-HIPEs presented a high extent of recovery, even after the five intervals back to 0.1 s^−1^. This can be beneficial for 3D printing as the restoration networks with a reversible structure breakdown are highly desired during extrusion printing. Specifically, E–SNC–N and E-SNP-N illustrated lower viscosity values at rest in addition to a higher drop in viscosity with time in the first, third, and five intervals. Such a drop in viscosity at a comparatively low shear rate (0.1 s^−1^) suggests that these inks were tremendously sensitive to the shearing force. Inversely, the viscosity values of E–SNC–M and E-SNP-M were less time-dependent sensitivity at rest in the first, third, and five intervals. This difference could be attributed to the formation of flocculated systems, where the droplets were closely arranged and aggregated to form a gel-like network structure.

The structural recovery was also studied by comparing the end viscosity values after conducting a time-dependent thixotropic response. In this case, the recovery percentage was obtained by measuring the end viscosity value of the fifth interval with the end viscosity value of the first interval. On comparing the recovery percentages, it can be found that control and E-SNP-N offered a weak structural recovery, specifying a poor elasticity with an unfitting stable structure. However, E–SNC–N presented a sensible recovery of about 66 %. On the other hand, the E–SNC–M and E-SNP-M showed brilliant structure recovery features of about 87 and 82 %, respectively, denoting a self-recovery behavior after exposure to high-level of shear rate. This highlights the development of a reversible network matrix in these inks with the restoration of their initial structures upon breakdown.

#### 3.2.3 Lissajous-Bowditch plots

**Fig. .5:**
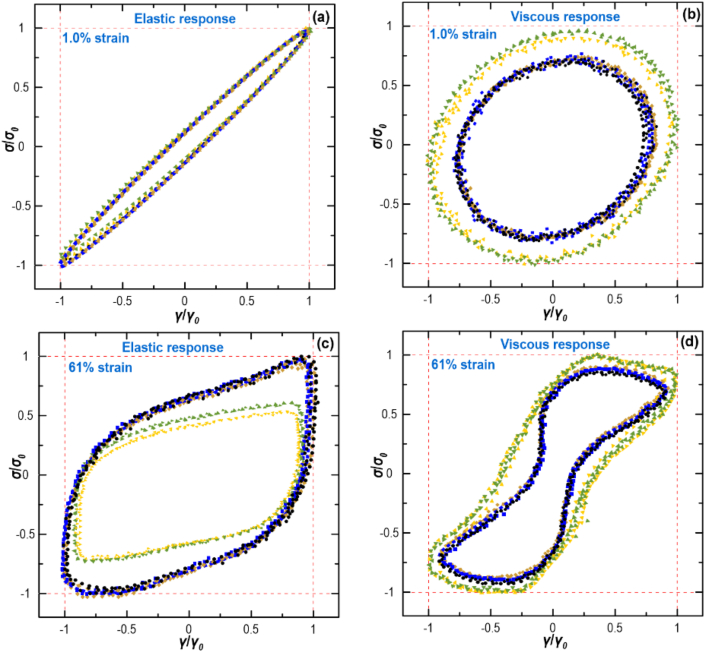
Normalized Lissajous–Bowditch plots of different Pickering-HIPEs. The results are shown for: Control (), E-SNP-N (), E-SNP-N () E-SNP-M (), and E–SNC–M ().

The linear viscoelastic parameters relate only to the first harmonic viscoelastic moduli (*G′*_1_ and *G″*_1_) of the stress response and show a vague physicomechanical meaning in the non-linear regions (which better represents the large deformation situation during 3D printing). However, it is vital to detect the visual difference in the non-linear stress response (*i.e.*, strains beyond the *LVR*) on the complex microstructure of materials, which is closer to the actual shearing force during the 3D printing process. For this reason, a complete oscillatory response at applied γ_0_ beyond *LVR* was evaluated (Hyun et al., 2011). The buildup of the droplet flocculation and consequent progress to the fluidized state in the Pickering-HIPEs and their local viscoelastic behaviors were examined through the normalized elastic or viscous Lissajous–Bowditch plots for two applied strain amplitudes of 1 and 61 %. The shapes of the Lissajous–Bowditch plots represent different microstructural responses under shear, with the area within the stress–strain and stress–strain rate loops being related to elastic and viscous energy, respectively. The non-linear character can be assessed through the magnitude and type of distortion of the Lissajous-Bowditch plots from an elliptical geometry (Fig. 5a-d).

The normalized ‘elastic’ and ‘viscous’ Lissajous plots of different Pickering-HIPEs are depicted in Fig. 5a,c and Fig. 5b,d, respectively, which provides valuable evidence of the structural evolutions upon large deformation. The ‘elastic’ Lissajous–Bowditch curves for all Pickering-HIPE variants at 1 % strain were elliptical in shape (Fig. 5a), showing a linear viscoelastic behavior. This is not strange, as the previously conducted strain sweep results (Fig. 4a) illustrated that the Pickering-HIPEs possessed a linear viscoelastic response within this range of strain magnitude. Once the strain was increased up to 61 %, the loops were altered from an ellipse to a parallelogram-like shape with distortion (Fig. 5c). This shows an eventual alteration from elastic- to viscous-dominated behaviors, denoting an enhanced viscous dissipation upon the intracycle deformation, in addition to extremely non-linear mechanical response. This highlights that the area inside the ‘elastic’ loop turns into a much larger parallelogram (Fig. 5c), while the area inside the ‘viscous’ loops decreases (Fig. 5d), proposing the physical properties of yielding behavior with the presence of a non-linear viscoelastic response. Compared to E–SNC–N and E-SNP-N, it is obvious that E-SNP-M and E–SNC–M offered somewhat less distortion from their original ellipse. This suggests that they possessed a more viscoelastic gel-like response (mainly elastic) with high stiffness.

Fig. 5b,d also depicts the shear stress–strain rate loops were gradually changed from the elliptical to a near *S*-shape after increasing strain from 1 to 61 %. This specifies a strong non-linear viscoelastic property with a high shear-thinning at the higher strain rates (Hyun et al., 2011). With increasing strain from 1 to 61 %, ‘viscous’ Lissajous plots of E-SNP-M and E–SNC–M also showed less distortion from their initial shape compared to the rest of Pickering-HIPEs. Once again, this case shows that these inks presented a more elastic solid-like feature beyond *LVR*, in agreement with the above-mentioned results of the ‘elastic’ curves.

The viscoelastic moduli beyond *LVR* denotes a relative alteration in mechanical strength of the Pickering-HIPEs on the basis of the strain amplitude, which distortions of the Lissajous–Bowditch curve are related to a greater harmonic contribution emerging inside a single cycle. Since FT-rheology simply measures poor signal during non-linear stress properties, to characterize a local deviation from linear viscoelastic stress inside the oscillation cycle, the Lissajous–Bowditch curves were converted into non-linear mechanical coefficient (*Q*). The *Q* curves as a function of strain amplitude of different HIPEs are illustrated in Supplementary Materials. With the increase in the strain amplitude, the *Q* gradually decreased, which exhibited a constant level (*Q*_0_) at the small amplitude of *γ* < 0.1 %. Compared to E–SNC–N and E-SNP-N, E-SNP-M and E–SNC–M showed an important reduction in the *Q*_0_ by approximately two orders of magnitude, though there was no difference in *Q*_0_ between all samples at a high amplitude of *γ* > 0.1 %.

### 3.3 Characterization of 3D printed objects

**Fig. 6:**
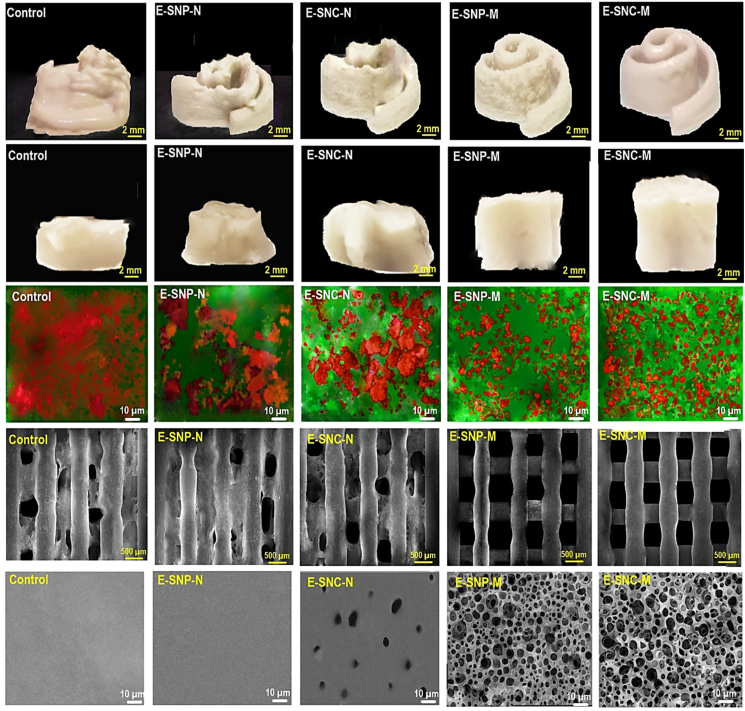
(Row *i*): The fresh (non-freeze-dried) 3D printed Torres, and (Row *ii*): fresh 3D printed Cubic. (Row *iii*): CLSM images of different non-freeze-dried 3D structures. (Row *iv*): Representative images of a one-layer of freeze-dried 3D printed-grid. (Row *v)*: FE-SEM images of freeze-dried 3D printed self-supporting scaffolds.

The prepared Pickering-HIPEs were used as a printing ink for the 3D printing process through a layer-by-layer deposition to manufacture different 3D structures (Fig. 6, Rows *i* and *ii*). The printing performance shows the integrity of the printing geometry and the clarity of the printed layers, presenting the quantitative basis to evaluate and quality control of 3D printed constructs. All Pickering-HIPEs were successfully extruded out from the nozzle tip due to some degree of shear-thinning (Fig. 4c). Specifically, the control sample revealed a poor printing quality with uneven 3D structures and high defects, leading to a higher susceptibility to collapse and cracking. Moreover, the printed layers of 3D shapes for control merged with each other (possibly due to a high height of the printed model), whose 3D structure spread over the surface. This may be attributed to the inferior viscoelastic property with poor gel-like structure, which is essential to support the printed layers (Shahbazi et al., 2022b). Similarly, E–SNC–N and E-SNP-N showed the sagging structure with a printed fusion layer and shape imprecision. They cannot uphold their structural integrity and endowed a low shape-fidelity. As already stated, these samples suffered from a low viscoelastic property with a poor thixotropy feature, which led to the development of a 3D structure with an unstructured geometry-retention feature. In contrast, E-SNP-M and E–SNC–M presented the printed objects with better printing quality, proposing improved stability and precise geometry (Fig. 6, Rows *i* and *ii*). This may be associated with their higher viscoelastic behavior and increased extent of nonlinear response under large deformations (Shahbazi et al., 2022b).

Fig. 6, Row *iii* also shows the microstructural observation of non-freeze-dried 3D printed objects obtained by CLSM. The control, E–SNC–N, and E-SNP-N presented a non-spherical shaped coalesced pocket/pool of oil. However, the CLSM images of E-SNP-M and E–SNC–M, by contrast, illustrated the oil droplets were homogenously distributed within the matrix. A similar microstructural property was detected in the previous publication (Dai et al., 2019), where the oil droplet offered a phase with spherical smooth shapes.

On the other hand, the freeze-dried printed-grids produced by E-SNP-M and E–SNC–M had excellent scaffold assembly with enhanced shape fidelity (Fig. 6, Row *iv*). In accordance with the results of viscoelastic properties (Fig. 4a and b), thixotropic behaviors (Fig. 4d), and non-linear stress response (Fig. 5) of their relevant inks, it was principally concluded that E-SNP-M and E–SNC–M inks endowed good viscoelasticity with excellent non-linear elastic characters, which led to brilliant printing performance after 3D printing (Shahbazi et al., 2021).

The printability index (*Pr)* relates to the development of a perfect square geometry (0 ≤ *Pr* ≤ 1) according to a printing pattern accuracy of an axial pore in an *XY* plane (Fig. 6 Row *iv*). In a specific grid geometry, the square shape shows a high precision with a *Pr* of 1. In this case, *Pr* < 1 presents a round shape and *Pr* > 1 denotes an irregular shape (Shahbazi et al., 2023b). Based on the quantification results, the *Pr* of E–SNC–N and E-SNP-N was found to be 1.22 ± 0.09 and 1.19 ± 0.13, respectively. While, E-SNP-M and E–SNC–M presented a *Pr* of 0.93 ± 0.12 and 0.98 ± 0.08, respectively. This reveals an outstanding printing quality for these samples. Furthermore, the pattern shape of the E–SNC–M was slightly rounded, as the *Pr* value was close to 1. Thus, the shape fidelity of E-SNP-M and E–SNC–M could be maintained well even if additional layers were deposited onto their structures.

Fig. 6 (Row *v*) also shows the microstructures of freeze-dried 3D printed self-supporting scaffolds. In this case, FE-SEM images showed a high-level of porosity concerning E-SNP-M and E–SNC–M scaffolds, which contained macropores, medium-sized pores, and small pores. While the large macropores originated via 3D printing, the hierarchically macroporous structures could be mainly induced by solvent evaporation. Here, oil droplets acted as the sacrificial template for macropores, which were developed upon the solvent removal (Shahbazi et al., 2023b). They also offered a uniform structure in terms of orientation and shape of spaces. Yet, the surface morphology of the freeze-dried 3D self-supporting scaffold regarding E–SNC–N and E-SNP-N was characterized by the lack of porous structure.

### 3.4 LCST-thermoresponsive properties of 3D printed constructs

**Fig. 7:**
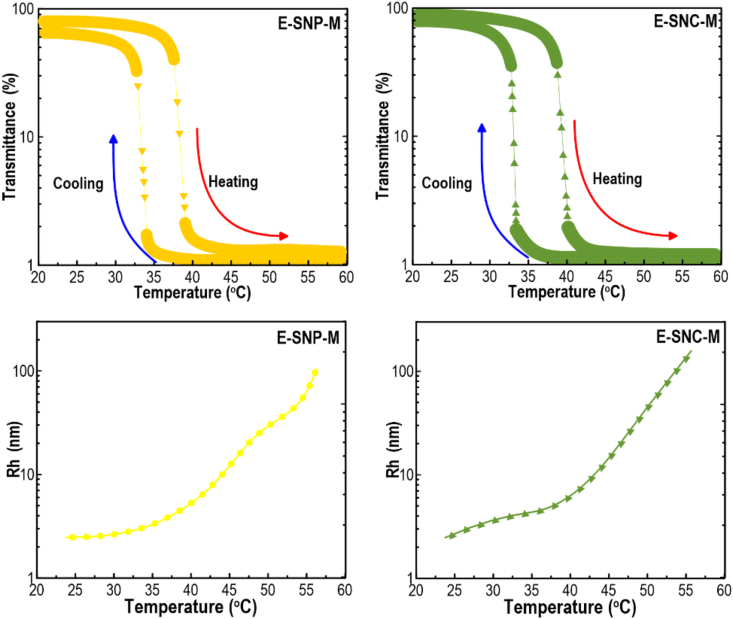
(Top): Transmittance curves illustrating the hysteresis between heating and cooling cycles. (Bottom): Temperature dependence of *R*_*h*_ for printed SNCs or SNFs samples (0.5 mg mL^-1^) at different temperatures.

Fig. 7 (Top) illustrates the transmittance curve of free-oil freeze-dried 3D structures concerning E-SNP-M and E–SNC–M as a function of temperature. As can be seen, there is a sharp decrease in transmittance with increasing temperature, whose LCST value was detected to be about 37 °C. Further, the thermal transition of the thermoresponsive 3D printed E-SNP-M and E–SNC–M was reversible. Unlike these samples, E–SNC–N and E-SNP-N did not exhibit a significant hysteresis (data not shown). This may be because of the lack of 1,2-butene oxide grafted onto their structure as it has been reported that this compound shows a unique thermoresponsive behavior (Zheng et al., 2019). The SNP-M and E–SNC–M exhibited a significant hysteresis, in which there was a pronounced hysteresis in the heating and cooling cycles as indicated by an approximately 5.0 °C temperature difference between the heating and cooling transmittance curves. Compared to the hysteresis observed in E-SNP-M, the hysteresis of E–SNC–M was a little higher. The hydroxybutyl-modified starch nanomaterials in the printed samples could be associated with developing more stable structures, separating hydrophobic parts from the hydrophilic phase as much as possible by intra- and intermolecular hydrophobic association. The developed intra- and intermolecular hydrophobics produce aggregation, which is prone to persist in the cooling phase (Shahbazi et al., 2024). With the increase in temperature, the modified starch nanomaterials became bigger and denser owing to an increase in the number and the size of hydrophobic aggregates. Once the temperature was consecutively decreased, the aggregations loosened and finally vanished, leading to a noticeable hysteresis. As a potential drug delivery system, these thermoresponsive macroporous 3D structures with LCST transition at body temperature (between 37 and 40 °C) can be used in the field of smart releasing of bioactive compounds. Furthermore, because of the amphiphilic nature of the 3D structure, both hydrophilic and hydrophobic medicines can be loaded into the 3D objects, in which a sustained drug-releasing process can be achieved with proper therapeutic efficacy.

Fig. 7 (Bottom) also shows the plot of the hydrodynamic radius (*R*_*h*_) of 3D structure variants in DMSO on the basis of temperature. In a lower temperature range (*T* < LCST), the *R*_*h*_ value was stabilized at a comparatively small value. Once the temperature was greater than LCST (*T* > LCST), the *R*_*h*_ value increased quickly during the heating phase. At a lower temperature, the starch chains in the modified SNCs or SNFs may exist in random coil conformation owing to the hydrogen-bonding interaction between their functional groups with DMSO molecules. When the temperatures increased to LCST, the chains would shrink into an entangled structure as the hydrogen bonds between the starch chains and DMSO molecules were clearly weakened or broken. When the temperatures reach higher ranges, the intra- and intermolecular hydrophobic associations are thermodynamically favored due to the presence of 1,2-butene oxide. Moreover, it is also possible some non-crystalline starches could be gelatinized without part of the intact crystalline and lamellar structures retained, which leads to an increase in *R*_*h*_. This production method might provide an interesting alternative to 4D printing of Pickering-HIPEs, in particular where the application necessitates a large pore structure with thermoresponsive features.

## 4 Conclusion

In conclusion, we offered a practicable paradigm to develop a 4D hierarchical macroporous thermosresponsive scaffold by 3D printing of Pickering-HIPE, which was stabilized by modified SNC and SNP to utilize given phase behaviors in colloidal dispersions. The interfacial and emulsifying behaviors of inks were evaluated through multiple techniques, such as strain or frequency sweeps, droplet sizes, thixotropy measurements, confocal imaging, and physical stability experiments. It was shown that the modification of starch nanoparticles could notably affect their interfacial features, producing a structured gel-like network formed by the flocculated oil droplets. Furthermore, flow behavior and thixotropy measurements showed that the modified starch nanoparticles produced shear-thinning and viscoelastic Pickering-HIPE-based inks with a large recovery rate, highlighting the brilliant structural recovery ability and the maintenance of the structure. Following 3D printing of Pickering-HIPEs, a highly porous structure with highly interconnected and open-cell matrices was fabricated, which possessed a thermoresponsive capacity. The results of this study offer the prospect of developing a thermoresponsive hierarchical porous structure by simply regulating the Pickering-HIPE formulation. In conclusion, the proposed tactics elucidated in this work hold promise in advancing its evolution and delivering extraordinary outcomes of 4D printed porous structures to food and pharmaceutical applications, especially the smart release of bioactive compounds.

## Funding

The research funding was provided by the University of Natural Resources and Life Sciences Vienna (BOKU).

## CRediT authorship contribution statement

**Mahdiyar Shahbazi:** Conceptualization, Methodology, Investigation, Collecting data, Validation, Data interpretation, Funding acquisition, Writing – original draft, Writing – review & editing. **Henry Jäger:** Writing – original draft, Writing – review & editing, Supervision. **Rammile Ettelaie:** Writing – original draft, Writing – review & editing. **Jianshe Chen:** Writing – original draft, Writing – review & editing. **Adeleh Mohammadi:** Methodology, Writing – original draft. **Peyman Asghartabar Kashi:** Investigation, Validation. **Marco Ulbrich:** Writing – original draft, Writing – review & editing.

## Declaration of competing interest

The authors declare that they have no known competing financial interests or personal relationships that could have appeared to influence the work reported in this paper.

## Data Availability

Data will be made available on request.
