# Supplementary material for: A smart thermoresponsive macroporous 4D structure by 4D printing of Pickering-high internal phase emulsions stabilized by plasma-functionalized starch nanomaterials for a possible delivery system
Source: Curr Res Food Sci. 2024 Feb 1;8:100686. doi: 10.1016/j.crfs.2024.100686 (PMC10878850; doi:10.1016/j.crfs.2024.100686)

A thermoresponsive macroporous structure by 4D printing of Pickering high internal phase emulsions stabilized by plasma-functionalized starch nanomaterials

*Mahdiyar Shahbazi ^a^* Henry Jäger* *^a^*, Rammile Ettelaie ^c^, Jianshe Chen^b^, Adeleh Mohammadi^d^, Peyman Asghartabar Kashi^e^, Marco Ulbrich*

^a^Institute of Food Technology, University of Natural Resources and Life Sciences (BOKU), Muthgasse 18, 1190, Vienna, Austria

^b^Food Colloids and Bioprocessing Group, School of Food Science and Nutrition, University of Leeds, Leeds, LS2 9JT, U.K.

^c^Food Oral Processing Laboratory, School of Food Science & Biotechnology, Zhejiang Gongshang University, Hangzhou 310018, China

^d^Faculty of Food Science and Technology, Gorgan University of Agricultural Sciences and Natural Resources, Gorgan 4913815739, Iran

^e^Faculty of Biosystem, College of Agricultural and Natural Resources, Tehran University, 31587-77871 Karaj, Iran

^f^Department of Food Technology and Food Chem., Chair of Food Process Engineering, Technische Universität Berlin, OfficeTK1 Ackerstraße 76, 13355 Berlin, Germany


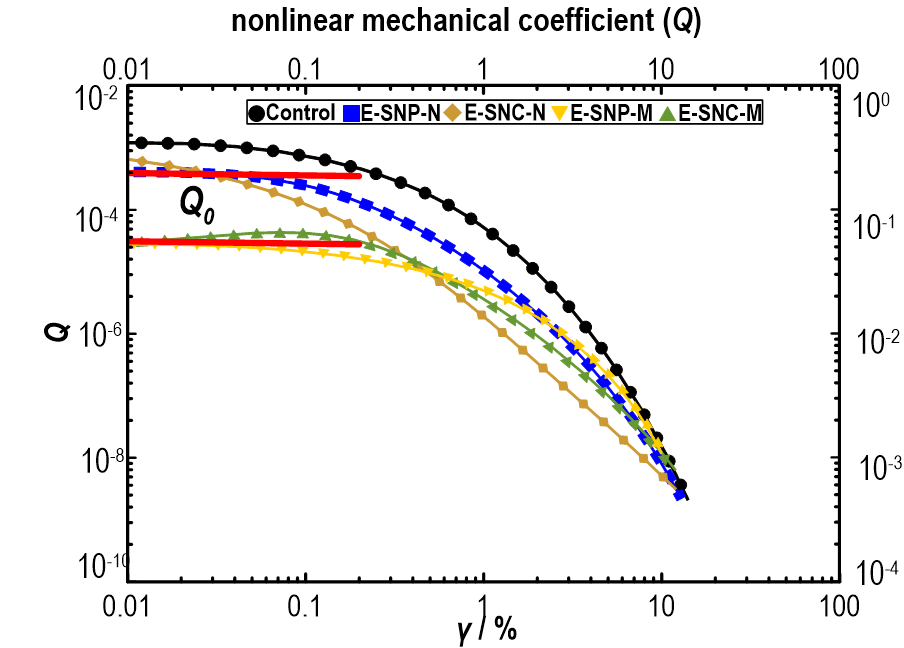

Supplement: Multimedia component 1 [file mmc1.docx]
